# Imaging clues for the diagnosis of various pathogenic causes of infectious spondylitis

**DOI:** 10.1007/s00256-025-04943-0

**Published:** 2025-05-16

**Authors:** Pornrujee Hirunpat, Theeraphol Panyaping, Wannisa Wongpipathpong, Siriporn Hirunpat

**Affiliations:** 1 Chakri Naruebodindra Medical Institute, Faculty of Medicine, Ramathibodi Hospital, Mahidol University, Samut Prakan, Thailand; 2 Division of Neurological Radiology, Department of Diagnostic and Therapeutic Radiology, Ramathibodi Hospital, Mahidol University, Bangkok, Thailand; 3 Department of Radiology, Faculty of Medicine, Prince of Songkla University, Hat-Yai, Songkhla, Thailand

**Keywords:** Imaging, CT, MRI, Infectious spondylitis, Spondylodiscitis

## Abstract

Infectious spondylitis is not a common disease; however, its incidence has increased recently due to the increasing number of older patients with chronic diseases and immunocompromised status globally. The clinical presentation of infectious spondylitis may be non-specific, causing delays in diagnosis and treatment, and leading to significant sequelae. Imaging usually plays a crucial role in characterizing the presence and extent of the disease, leading to proper management, reduced mortality, and long-term neurological morbidity. Many studies have proposed imaging features to distinguish between the common causes of infectious spondylitis, pyogenic or tuberculous infections, while the less common infections, including those caused by fungi or other bacterial organisms such as brucellosis, melioidosis, and actinomycosis, are believed to lack specific imaging characteristics. In this review, we highlight the characteristic imaging findings of both common and uncommon pathogens, which can serve as key clues for accurately diagnosing various pathogenic causes of infectious spondylitis.

## Introduction

Although infectious spondylitis is a relatively rare cause of back pain, its incidence has increased in recent years owing to the globally increasing number of older patients with chronic diseases such as diabetes mellitus, renal failure, conditions requiring steroid or other immunosuppressive therapies, and other immunocompromised conditions [1, 2]. However, its clinical presentation may be non-specific, causing delayed diagnosis and treatment, and leading to significant sequelae. Imaging plays a crucial role in characterizing the presence and extent of the disease, facilitating proper management, reducing mortality, and minimizing long-term neurological morbidity.

Many studies have proposed imaging features to distinguish between the common causes of infectious spondylitis, such as pyogenic and tuberculous infections; however, less common infections, including those caused by parasites, fungi, or other bacterial organisms, are believed to lack specific imaging characteristics. In this article, we review and emphasize the characteristic imaging findings of common and uncommon pathogens that can provide clues for the accurate diagnosis of various pathogenic causes of infectious spondylitis.

## Nomenclature and epidemiology

Spondylitis refers to the inflammation of the spine: when caused by an infection, it is termed infectious spondylitis, which is a serious cause of back pain and accounts for 2–5% of all osteomyelitis cases [1, 3, 4].

Infectious spondylitis can affect one or more spinal structures, including the vertebrae (osteomyelitis), intervertebral discs (discitis), or facet joints (septic arthritis) [4, 5]. When the infection is more extensive, affecting both the vertebrae and intervertebral discs, it is referred to as spondylodiscitis or discitis-osteomyelitis [3]. However, some sources may use these terms interchangeably.

Although more common in older patients, infectious spondylitis follows a bimodal distribution, with incidence peaking in individuals under 20 years and between 50 and 70 years. It exhibits a male-to-female predominance ratio of 1.5–2:1, particularly in older populations due to a higher frequency of comorbidities in men over 60 years of age [1].

## Pathophysiology and imaging characteristics

Most cases of infectious spondylitis result from the hematogenous spread of septic emboli, which may originate from the urinary tract, rectosigmoid colon, pulmonary, or skin/soft tissue infections [2, 4]. This spread can occur through the arterial or venous systems, with the arterial route being more common [1, 4, 6–8]. Arterioles branching from the arteries terminate at the anterior subchondral region, where infection often begins [6]. The vertebral venous system, including Batson’s paravertebral venous plexus, can also introduce pathogens from remote sites into the spine through retrograde flow owing to its valveless system [1, 7]. Other less common causes include direct inoculation from spinal surgery, trauma, or nearby infected soft tissues [1, 4, 7]. However, imaging findings of infectious spondylitis vary between children and adults.

In adults, intradiscal anastomoses regress over time, and the intraosseous arteries function as end arteries [3, 6]. The deposition of septic emboli in these end arteries leads to ischemia and infarction in the vertebral endplates, particularly on the anterior side, resulting in bone destruction and collapse [1, 3, 6]. Additionally, the intervertebral disc becomes nearly avascular in adulthoods and lacks a defense mechanism against infection, which allows the infection to spread from one endplate to an adjacent vertebral endplate [3]. This progression is characteristic of infectious spondylodiscitis.

**Fig. 1:**
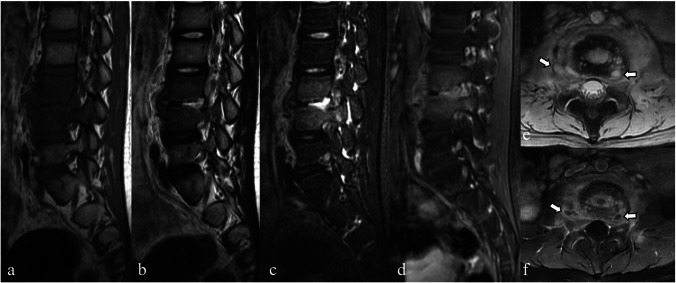
Magnetic resonance images of a 9-year-old female patient with infectious spondylitis. Sagittal T1-weighted (**a**), T2-weighted (**b**), short tau inversion recovery (STIR) (**c**), and fat-suppressed contrast-enhanced T1-weighted (**d**) images demonstrate destroyed L3–L4 intervertebral discs, characterized by disc space narrowing, hyperintense T2-weighted/STIR signals, and enhancement. Despite edematous marrow of the surrounding L3 and L4 vertebral bodies seen as hypointense areas on the T1-weighted image and hyperintense areas on the T2-weighted/STIR images with mild diffuse enhancement, no significant bone destruction or collapse are noted. Axial T2-weighted (**e**) and fat-suppressed contrast-enhanced T1-weighted (**f**) images show prominently thickened soft tissue in the entire perivertebral region and small abscesses within the intervertebral disc and right para-vertebral region (arrows)

In children, intradiscal anastomoses remain open, providing greater blood supply and fewer end-arteries at the endplates than those in adults, which helps protect the vertebral body from infarction caused by septic emboli [3, 6, 9]. The intervertebral disc remains vascularized, increasing susceptibility to primary discitis (Fig. 1) [1, 3, 6, 7, 9]. This vascularization also limits the spread of infection from the endplate to adjacent vertebrae [1, 3]. The extensive intraosseous vascular network gradually shrinks and disappears by the age of 7–13 years [5, 7]. However, in older patients with disc degeneration, revascularization may occur through radial tears in the annulus fibrosus, which can lead to primary discitis [6, 7].

## Pyogenic spondylitis

The term “pyogenic” refers to the production of pus. Therefore, pyogenic spondylitis is a pus-producing spinal infection caused by bacteria. However, not all bacteria cause pyogenic spondylitis.

*Staphylococcus aureus* is the most common causative agent of pyogenic spondylodiscitis, accounting for over 75% of cases [1, 2, 5, 7]. Certain microorganisms are associated with specific risk factors. For instance, *Streptococcus* is common in patients with endocarditis, with one-third of pyogenic spondylitis cases having endocarditis and approximately 2–20% of patients with endocarditis developing pyogenic spondylitis [1]. *Salmonella* is usually observed in patients with sickle cell disease or other immunocompromised conditions, such as leukemia, diabetes, or prolonged steroid use [3, 10, 11]. *Pseudomonas aeruginosa* is frequently found in intravenous drug users [2, 5, 7]. *Klebsiella pneumoniae* is commonly found in alcohol abusers, and *Escherichia coli* is commonly linked to urinary tract infections [5].

Pyogenic spondylodiscitis predominantly affects the lumbar spine (60%), followed by the thoracic spine (30%) and cervical spine (10%). The thoracolumbar predominance is attributed to the high frequency of urinary tract and pelvis infections [4]. The arterial pathway serves as the most common route for infection, thereby making the anterior subchondral aspect of the vertebral bodies the primary site of initial infection [2, 4]. Disc involvement typically occurs early in the disease course due to the action of proteolytic enzymes [5]. Pyogenic infections usually affect a single vertebral segment, involving two adjacent vertebral bodies and the intervertebral disc between them [4].

The symptoms of pyogenic spondylodiscitis typically manifest insidiously and appear earlier than those of tuberculous infections. Therefore, imaging studies are often required early in the disease course. Plain radiographs have poor sensitivity and specificity for diagnosing spinal infections and may appear completely normal in the early phase. Detecting bone loss on plain radiographs requires up to 30–40% reduction in bone matrix. Therefore, plain radiographic changes in infectious spondylitis are generally not apparent until 2–8 weeks after symptom onset. After 8–12 weeks, subchondral radiolucency, endplate irregularities, loss of disc height, and bone destruction may become visible and progressively pronounced. In cases of chronic infection, spinal deformities such as kyphosis and scoliosis become apparent after approximately 4 months [1]. Endplate sclerosis and ankylosis of the disc space may also be observed in patients undergoing treatment or in the later reparative phase [2].

Computed tomography (CT) images can provide better details than plain radiographs and allow better assessment of bony abnormalities, such as endplate and vertebral body erosion. Additionally, contrast-enhanced CT is superior to non-contrast study since it can provide better detection of epidural, paravertebral inflammatory tissue, and abscesses. However, CT may fail to detect or accurately determine the extent of spinal cord or nerve root involvement and the findings may also be normal within the first 3 weeks [1]. Despite these limitations, CT is still a good tool for guiding aspiration or biopsy, aiding in accurate diagnosis and treatment planning [2, 7].

MRI is the modality of choice for evaluating spinal infections [1, 2]. Contrast-enhanced MRI has a sensitivity of 97% (specifically 93%) and an accuracy of 94% for diagnosing infectious spondylitis [1]. It is the most excellent tool for evaluating disease extent, not only in the bone but also in the disc, surrounding soft tissue, spinal cord, and nerve roots, which are crucial for planning surgical approaches.

**Fig. 2:**
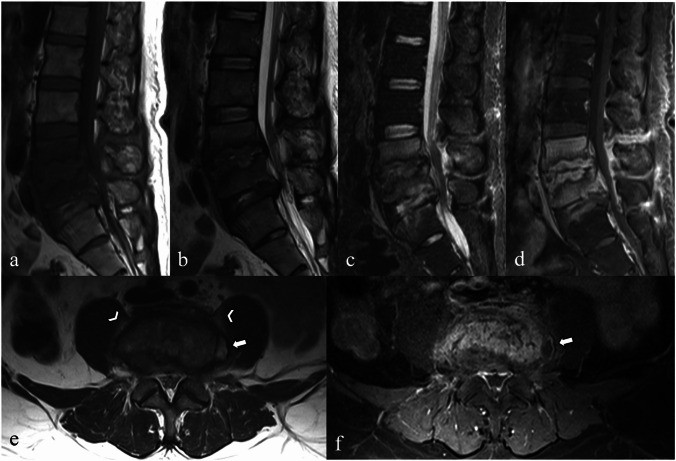
Magnetic resonance images of a 37-year-old male with pyogenic spondylitis caused by *Streptococcus agalactiae*. Sagittal T1-weighted (**a**), T2-weighted (**b**), short tau inversion recovery (STIR) (**c**), and fat-suppressed contrast-enhanced T1-weighted (**d**) images show hypointense T1-weighted and hyperintense T2-weighted/STIR signals with enhancement of the severely destroyed L4-L5 intervertebral disc, partially destroyed L5-S1 disc, and the surrounding L4, L5, S1 endplates. Edematous, enhancing marrow involving the entire L4 and L5 vertebral bodies is also noted. Axial T2-weighted imaging at the L4–L5 level (**e**) demonstrates the imaging psoas sign, characterized by T2 hyperintensity within the bilateral psoas muscles (arrowheads). Axial fat-suppressed contrast-enhanced T1-weighted image (**f**) shows enhancement of the perivertebral inflammatory soft tissue and a small abscess in the left paravertebral region (arrows)

Typical MRI findings include hypointensity on T1-weighted images, hyperintensity on T2-weighted images of the vertebral bodies and disc indicating edema, and irregularities of the vertebral endplates (Fig. 2) [4, 7]. In the early stages, vertebral body destruction is usually limited to the endplates. The hyperintensity on T2-weighted images with enhancement of the infected disc and surrounding vertebral endplates aids in its differentiation from degenerative discs [2, 4]. The loss of intranuclear cleft, a low signal intensity band on T2-weighted images in noninfected normal discs, is not helpful, as it appears in both infectious spondylitis and degenerative disc disease [1].

Spinal infections can extend into the epidural or paraspinal spaces, leading to the formation of inflammatory soft tissue/mass or abscesses. The epidural space is particularly prone to infection owing to its rich venous plexus. Pyogenic paraspinal abscesses are typically small with thick, irregular, rim-enhancing walls. Diffusion-weighted imaging (DWI) is also a valuable tool for detecting abscesses or pus in the spinal or paraspinal regions due to its strong restriction of water diffusion [1, 5, 6].

**Fig. 3:**
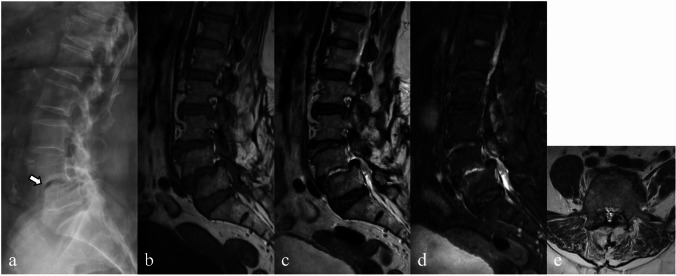
Magnetic resonance images of a 72-year-old female patient with recurrent back pain and a history of decompression surgery performed 20 years ago. Conventional radiograph (lateral view) (**a**) showing lumbar spondylosis and L4–L5 spondylolisthesis with L4–L5 intradiscal vacuum phenomenon (arrow). Sagittal T1-weighted (**b**), T2-weighted (**c**), and short tau inversion recovery (STIR) (**d**) images show L4–L5 spondylolisthesis with hyperintense T2-weighted/STIR signals in the degenerative disc resembling infectious spondylitis. A relatively preserved dark line of cortical endplate is detected on both T1-weighted and T2-weighted images. An axial T2-weighted image (**e**) also reveals no abnormal paravertebral or epidural soft tissue to suggest infectious spondylitis. No infection was evident on clinical and follow-up imaging after 2 years

Although rare, the presence of intradiscal fluid in degenerative discs on MRI images may raise the suspicion of infectious spondylitis. This finding could be due to a fissure in the degenerative disc or related to the intradiscal vacuum phenomenon and Modic type 1 degenerative endplate changes (Fig. 3). The vacuum phenomenon is a common finding in degenerative discs but is rarely seen with infection [12].

Differentiating pyogenic spondylitis from endplate degenerative changes is important because the treatments are completely different. Imaging clues suggest infection rather than degenerative changes, including the presence of cortical endplate erosion/destruction, which may be more easily identified on T1-weighted images, and the presence of abnormal paraspinal soft tissue [4, 6]. Hyperintensity of the psoas muscle a T2-weighted image (psoas sign) is also a useful clue (Fig. 2) [13].

Although DWI is a valuable tool for detecting abscesses or pus in spinal or paraspinal regions, Modic type 1 endplate changes in degenerative disc disease can also exhibit diffusion abnormality mimicking infection. However, the typical well-defined, paired band-like regions of restricted diffusion on DWI images, affecting two contiguous vertebral bodies on sagittal images—known as “the claw sign”—is highly suggestive of degenerative disc disease and serves as a useful feature for differentiating it from discitis/osteomyelitis [1, 14].

## Tuberculosis

Tuberculous spondylitis, also known as Pott disease, is caused by *Mycobacterium tuberculosis*, a Gram-positive acid-fast bacillus [2, 15]. This infection commonly affects the thoracic spine and tends to be indolent, with a gradual onset of symptoms over months to years, leading to a late diagnosis [2, 5, 7, 15, 16].

Hematogenous spread can occur via arteries or veins, resulting in various patterns of tuberculous infection. In arterial spread, the infection typically originates in the anterior subchondral vertebra and spreads subligamentously beneath the anterior or posterior longitudinal ligament, leading to the involvement of multiple vertebral segments [8, 16]. Through the venous route, tuberculosis infections exhibit a higher propensity for posterior element involvement compared to pyogenic infection [1, 3, 4, 6–8, 14].

Unlike pyogenic spondylitis, tuberculosis spondylitis lacks proteolytic enzymes to break down the vertebral disc, resulting in the relative preservation of the disc space [1, 2, 4, 5, 16–18]. This may also explain why the infection typically spreads through the paravertebral soft tissue in an anterolateral direction [16] surrounding the disc rather than directly penetrating it.

**Fig. 4:**
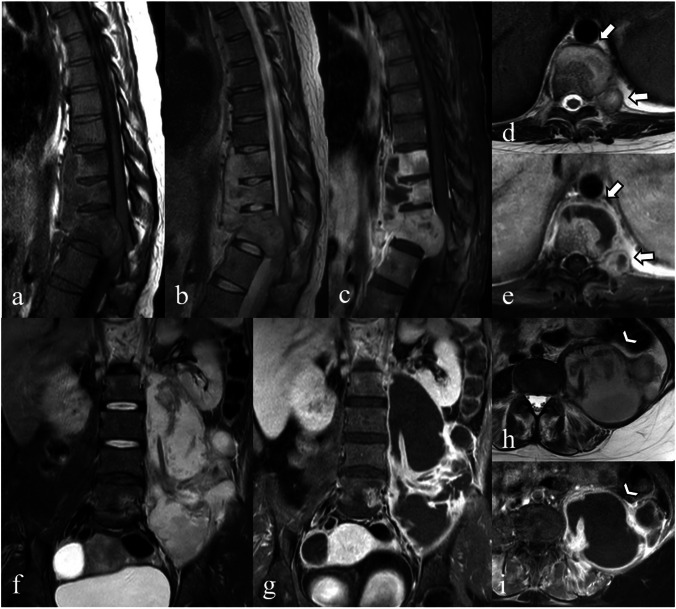
Magnetic resonance images of a 29-year-old female patient with tuberculous spondylitis. Sagittal T1-weighted (**a**), T2-weighted (**b**), and fat-suppressed contrast-enhanced T1-weighted (**c**) images show contiguous destruction of T9–T12 vertebrae with severely collapsed T11–T12 vertebral bodies causing severe kyphotic angulation (gibbus deformity). Anterior subligamentous spreading, intraosseous abscess, and epidural abscess with conus medullaris compression are also noted. Axial T2-weighted (**d**) and fat-suppressed contrast-enhanced T1-weighted (**e**) images show intraosseous and left paravertebral abscesses (arrows in **d** and **e**). Coronal T2-weighted (**f**), fat-suppressed contrast-enhanced T1-weighted (**g**), axial T2-weighted (**h**), and fat-suppressed contrast-enhanced T1-weighted (**i**) images also show large left paravertebral abscess along the left psoas muscle (psoas abscess), extending to left-sided pelvic cavity (arrowheads in **h** and **i**)

Therefore, the classic imaging findings of tuberculous spondylitis (Fig. 4) include sparing of the intervertebral disc in the early stages, posterior element involvement, subligamentous spread, heterogeneous vertebral body enhancement patterns, and involvement of multiple vertebral bodies at three or more levels [1, 2, 4, 15, 17, 18].

As the disease progresses, further vertebral destruction can lead to extensive loss of vertebral height, severe kyphotic angulation (gibbus deformity), and the formation of disc and paravertebral inflammatory soft tissue masses and abscesses, which often spread anterolaterally [1, 2, 4, 7, 15, 17, 18]. Abscesses in tuberculous spondylitis are often large, with thin, smooth, rim-enhanced walls, and they may involve the vertebra (intraosseous abscess). They can also be calcified, differing from the thick irregular non-calcified wall, and may involve discs being affected by pyogenic abscess [8, 18]. Calcification within a paravertebral abscess, particularly when seen in conjunction with other imaging features, such as vertebral body destruction, disc space narrowing, and spinal deformity, is a hallmark of tuberculous spondylitis.

Despite the availability of several imaging features that may aid in distinguishing between pyogenic and tuberculous spondylitis, differentiation between the two entities is often a diagnostic challenge.

**Fig. 5:**
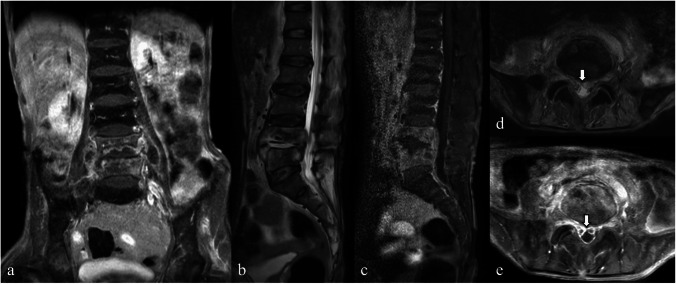
Magnetic resonance images of a 79-year-old male patient with tuberculous spondylitis. Coronal fat-suppressed contrast-enhanced T1-weighted (**a**), sagittal T2-weighted (**b**), fat-suppressed contrast-enhanced T1-weighted (**c**) images show tuberculous spondylodiscitis with perivertebral inflammatory soft tissue and anterior epidural abscesses. Axial T2-weighted (**d**) and fat-suppressed contrast-enhanced T1-weighted (**e**) images reveal preservation of the anterior meningovertebral ligament resulting in a bilobed appearance of anterior epidural abscess (arrows)

In the case of anterior epidural abscess, the preservation of the anterior meningovertebral ligament—a septum anchoring the posterior longitudinal ligament to the periosteum—suggests tuberculous spondylitis with high sensitivity (83.3%) and specificity (100%). This preservation is hypothesized due to the absence of proteolytic enzymes (Fig. 5) [15].

## Other less common bacterial infection

### Brucellosis

Brucellosis is a zoonotic infection caused by gram-negative bacilli of the genus Brucella that are commonly transmitted through the consumption of contaminated milk or meat [19–23]. Brucellosis occurs worldwide, with the highest rates observed in the Mediterranean region, Middle East, South Asia, and Central and South America [19, 20, 22, 24]. Musculoskeletal infections are a common manifestation of brucellosis, with the spine being the most frequently affected site [20, 25]. Like tuberculosis, brucellosis can cause granulomatous inflammation [20]. Identifying the MRI features of this type of infectious spondylitis can be helpful, as biopsies and blood cultures often yield negative findings [1]. Moreover, inflammatory syndrome (clinically and biologically) in patients with brucellar spondylitis is less frequent [1].

**Fig. 6:**
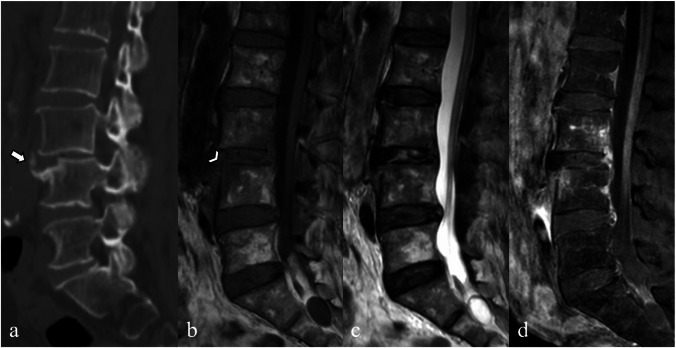
A sagittal computed tomography image (**a**) of the lumbosacral spine of a 70-year-old female patient with brucellar spondylitis. Bone erosion is observed at the anterosuperior corner of the L4 vertebral body, termed the “Pedro Pons’ sign,” along with reactive bone sclerosis with anterior osteophytes that form a “parrot’s beak” appearance (arrow). Sagittal T1-weighted (**b**), T2-weighted (**c**), and fat-suppressed contrast-enhanced T1-weighted (**d**) images show focal bone destruction at the anterosuperior endplate of L4 vertebral body (arrowhead in image **b**) with enhancement of the opposing endplate of L3–L4 vertebral bodies and mild hyperintense T2-weighted signal changes of the intervertebral disc

Brucellar spondylitis mostly occurs in the lumbar region, particularly at the anterosuperior corner [1, 4, 19, 20, 22, 24–30]. A key characteristic of brucellosis is the simultaneous onset of new bone formation and bone destruction, which explains why brucellosis spondylitis tends to preserve the normal vertebral architecture or cause only mild deformities [4, 19, 25, 28, 30]. Gibbus deformities are therefore rare [22, 29, 31]. On plain radiographs and CT scans, key findings include bone erosion at the anterosuperior corner of the vertebral body, termed the “Pedro Pons’ sign” [16, 32], and perivertebral osseous construction, resembling anterior osteophytes with “parrot’s beak” appearance (Fig. 6) [20, 33, 34]. However, distinguishing these features from those of degenerative diseases can be challenging without MRI [21, 23, 27, 29–31].

Brucellosis spondylitis can manifest in two distinct forms, focal and diffuse, each with unique characteristics that affect the course of the disease [33, 34]. In the focal form (Fig. 6), lesions primarily affect the discovertebral junction, located mainly in the anterosuperior region of the vertebral body, with limited spread. This pattern likely begins with sufficient blood supply to the upper endplate and progresses non-invasively. Associated features such as bony sclerosis, parrot’s beak appearance, and small gas pockets, which are possibly due to soft tissue destruction, may also be observed in this form. Typically, the disc and surrounding soft tissues remain unaffected.

**Fig. 7:**
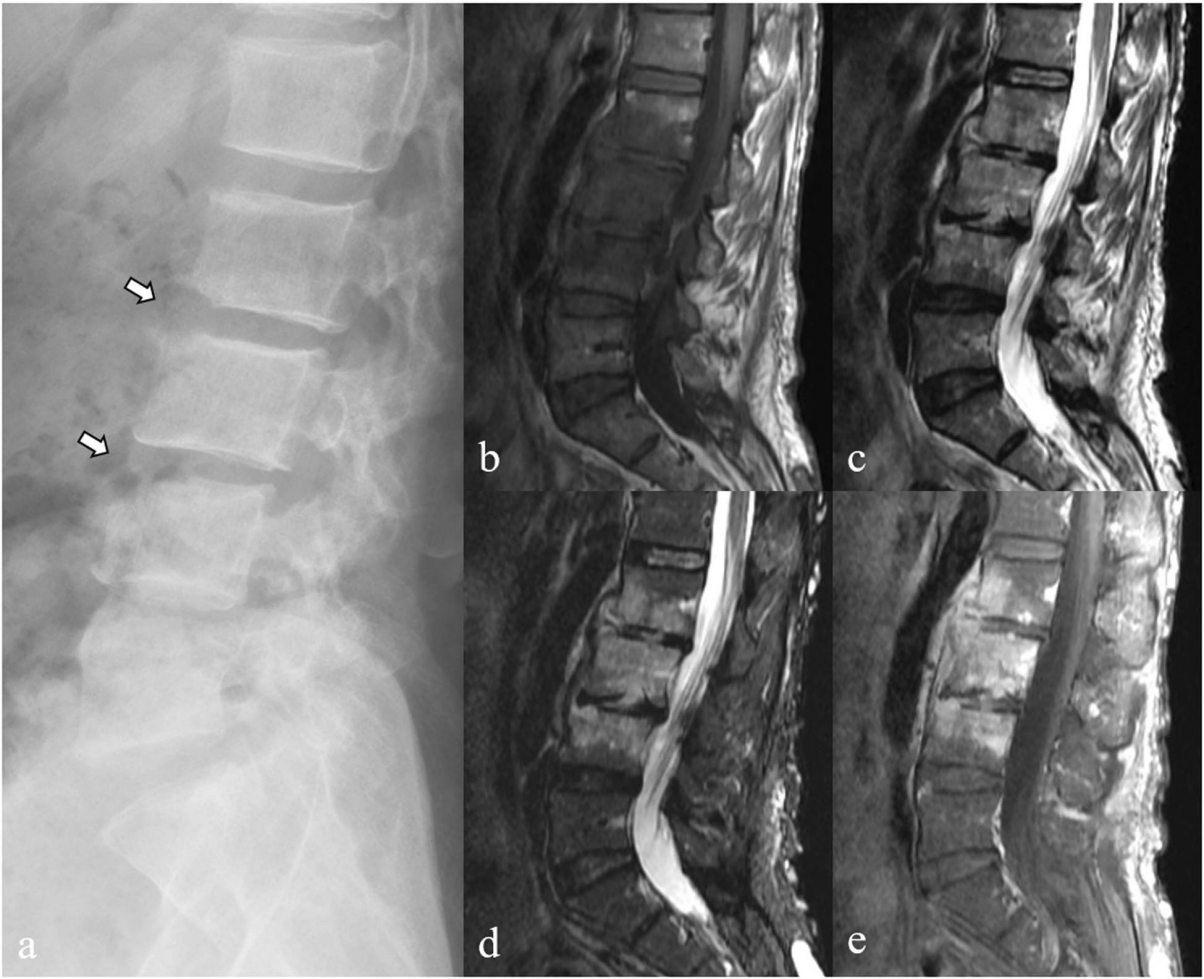
A case of diffuse brucellar spondylodiscitis in a 56-year-old female patient with underlying type I neurofibromatosis. A lateral plain radiograph (**a**) shows erosion of the anterior opposing endplates at L2–L3 to L3–L4 levels (arrows) with a few small anterior osteophytes. Sagittal T1-weighted (**b**), T2-weighted (**c**), short tau inversion recovery (STIR) (**d**), and fat-suppressed contrast-enhanced T1-weighted (**e**) images show hypointense T1-weighted and hyperintense T2-weighted/STIR signals with enhancement of L2–L4 vertebral bodies and intervening discs, which reveal the absence of nuclear clefts

In the diffuse form, lesions involve the entire vertebra and spread to adjacent vertebrae through ligament and vascular connections (Fig. 7). In some instances, these lesions may extend into the intervertebral disc, potentially resulting in the formation of Schmorl’s nodes. In severe cases, the disease can lead to the development of epidural and paravertebral abscesses, most of which are small, with thin and irregular walls [19].

### Melioidosis

Melioidosis is an infectious disease caused by *Burkholderia pseudomallei*, a gram-negative bacillus commonly found in muddy soil, surface water, and plants [35–41]. The endemic regions include Southeast Asia, mostly the northeastern provinces of Thailand, and northern Australia [35–39]. Recent literature suggests an additional endemic in South Asian countries, such as India, Sri Lanka, and Bangladesh [40, 41]. Melioidosis spreads through the ingestion of contaminated materials or inoculation via open skin [36, 39]. Individuals with diabetes, a history of alcohol use disorder, or immunocompromised conditions have an elevated risk of contracting this infection [35–39, 41].

**Fig. 8:**
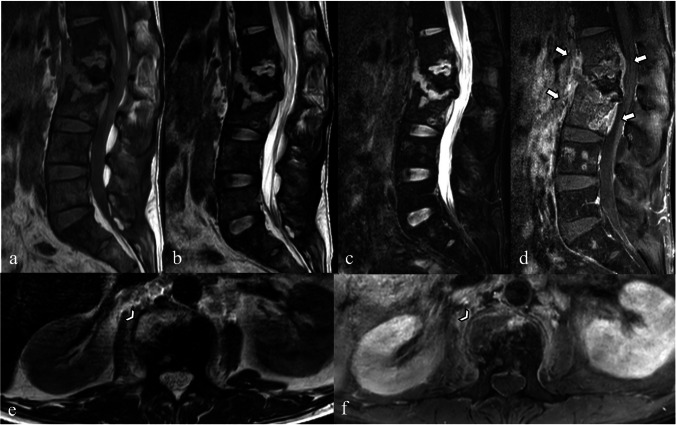
Magnetic resonance images of a 40-year-old male patient with melioidosis spondylodiscitis that mimics tuberculous infection. Sagittal T1-weighted (**a**), T2-weighted (**b**), short tau inversion recovery (**c**), and fat-suppressed contrast-enhanced T1-weighted (**d**) images show partially destroyed L1–L3 vertebrae, severely collapsed L2 vertebra with anterior subligamentous spread, and peri-vertebral and anterior epidural soft tissue involvements (arrows in **d**) causing moderate central spinal canal stenosis. Axial T2-weighted (**e**) and fat-suppressed contrast-enhanced T1-weighted (**f**) images demonstrate a small right paravertebral abscess (arrowheads)

Despite its rarity, melioidosis can lead to infectious spondylitis, characterized by altered signal intensity in the vertebrae and intervertebral discs, often accompanied by epidural and paravertebral collections [38, 40]. The lumbar region is the most frequently affected area [40]. Previous reports [36, 40, 42–45] have described manifestations of melioidosis that closely mimic tuberculous infections, making diagnosis and treatment more challenging. Thus, patients with imaging findings consistent with spinal tuberculosis, especially in endemic regions and with relevant risk factors, should be suspected of having a melioidotic infection (Fig. 8).

### Actinomycosis

Actinomycosis is an infection caused by gram-positive, branching, acid-fast negative, anaerobic bacteria [46, 47]. The most common species found in humans is *Actinomyces israelii* [47–49]*.* These bacteria are often found in the normal flora of the oropharynx and vaginal tract [46, 47, 50, 51]. The risk factors include poor oral hygiene, dental procedures, and use of intrauterine or intravaginal devices [46, 47, 50, 52, 53]. Actinomycosis can occur in both immunocompromised and immunocompetent individuals, with the latter being more prone to spinal abscesses and osteomyelitis [46].

The disease course is chronic and characterized by suppurative and granulomatous inflammation, abscess formation, and draining sinuses that may express sulfur granules [47–49]. These bacteria are not highly virulent and require mucosal or cutaneous disruption to penetrate healthy tissue [47, 48, 52, 53]. Actinomycosis most commonly affects the cervicofacial region, particularly at the angle of the jaw, followed by the thoracic and abdominal regions, skin, and brain [48, 49, 52].

**Fig. 9:**
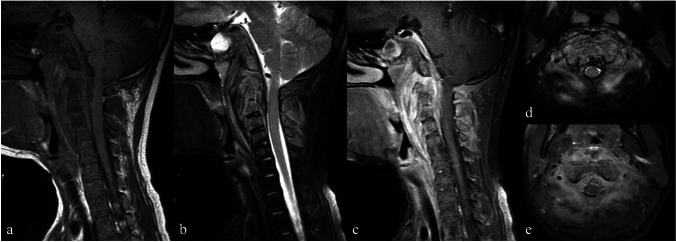
Magnetic resonance images of an 11-year-old female patient with actinomycosis spondylitis involving the cervical spine. Sagittal T1-weighted (**a**), short tau inversion recovery (**b**), fat-suppressed contrast-enhanced T1-weighted (**c**), axial T2-weighted (**d**), and contrast-enhanced T1-weighted (**e**) images show diffuse enhancement of C1–C5 vertebrae, with thickened and enhanced soft tissue at perivertebral and anterior epidural regions. The cervical discs are preserved without gross destruction. Diffuse enhancement of the clivus, representing skull base osteomyelitis, along with retroclival, epidural enhanced soft tissue and reactive fluid; partially opacified sphenoid sinus is also noted

Spinal infections caused by actinomycosis are rare, with only a limited number of cases reported [46–57]. This infection typically extends from adjacent soft tissue infections (Fig. 9) [52]. Kim and Kim [46] reported a direct connection between retropharyngeal and epidural abscesses in patients with cervical actinomycosis. Other routes of infection include hematogenous spread from distant sites [52, 55].

Contrary to other infectious spondylitis, actinomycosis usually spares the discs [49, 50, 55]. Due to the slow nature of the disease, bone destruction typically occurs with new bone formation, explaining the uncommon findings of vertebral collapse and angular deformities [49, 50, 57].

### Fungal infection

Fungal spinal infection, an opportunistic infection, is much less common than pyogenic and tuberculous infection, and accounts for approximately 0.5% of all cases [1]. However, they are becoming more common owing to the increasing number of individuals with immunocompromised conditions, including HIV, diabetes, post-transplantation, and ongoing chemotherapy or immunosuppressive therapy [4, 10]. The most common fungal pathogens implicated in humans are *Aspergillus* and *Candida* species [1, 4, 10, 58, 59]. The radiologic features of fungal infections lack specificity [1]. Early diagnosis of fungal spondylitis is therefore difficult and relies on high suspicion and clinical judgement, particularly in patients with immuno-compromised conditions.

Despite the lack of specific imaging features, many studies have characterized radiological features of fungal spondylodiscitis and have proposed distinct imaging patterns to differentiate it from other infections. Previous studies [10, 60, 61] suggest that fungal infections typically follow an indolent course, with less osseous destruction and subtle MRI changes, including mild T1 hypointensity, T2 hyperintensity, and slight contrast enhancement. These changes are likely due to mild inflammation caused by a compromised immune system. Williams et al. [58] reported a case with minimal to moderate paraspinal inflammation, unlike tuberculosis, which typically presents with a larger inflammatory component. Additionally, the lack of disc hyperintensity on T2-weighted images and preservation of intranuclear cleft are signs suggestive of fungal infection, which may aid in its differentiation from pyogenic spondylitis [16, 58, 61]. However, these imaging findings are relatively qualitative and subjective which can be observed in the early stage infectious processes. As the disease progresses, fungal spondylodiscitis may present with more pronounced MRI findings, and the severity of these findings may vary depending on the patient’s immune status, making differentiation more challenging.

Candida spondylitis commonly affects the lumbar spine with posterior element involvement, while preserving the disc, similar to that in tuberculosis [5, 60]. According to Lee et al. [59], candida spondylitis can be suspected when infectious lesions contain low-signal spinal inflammatory masses on T2-weighted imaging with small paraspinal abscesses in patients with immunocompromised conditions. This is unlike pyogenic or tuberculosis infections, which typically display high signal hyperintensity on T2-weighted images.

**Fig. 10:**
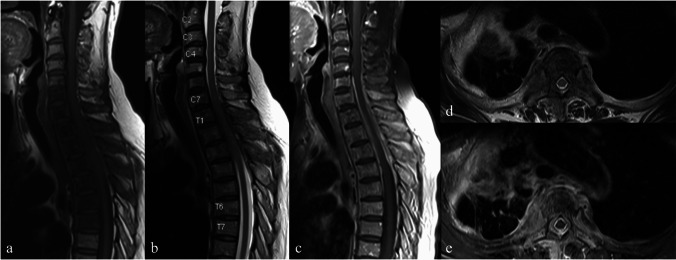
Magnetic resonance images of a 64-year-old female patient with a history of old pulmonary tuberculosis, which later developed into aspergillus spondylitis. Sagittal T1-weighted (**a**), sagittal T2-weighted (**b**), and fat-suppressed contrast-enhanced T1-weighted (**c**) images reveal multiple vertebral involvement from C5 to T7, with subligamentous spreading and perivertebral and epidural soft tissue compressing the cervical spinal cord. Axial T2-weighted (**d**) and fat-suppressed contrast-enhanced T1-weighted (**e**) images at the T3-T4 level, bilateral paraspinal involvement, more prominent on the right side which also involves adjacent pleura, right apical lung, and right rib are also demonstrated

Kwon et al. [62] suggested that irregularities or serrated margins of the vertebral endplates, along with subchondral T2 hypointensity, indicate the possibility of aspergillus spondylitis. Subchondral T2 hypointensity is caused by paramagnetic and ferromagnetic elements in fungi, similar to fungal sinusitis observed on T2-weighted images. Additional findings include the involvement of multiple vertebral segments with skip lesions or subligamentous spreading (Fig. 10), which are often mistaken for tuberculous spondylitis. In addition, thick, irregularly walled abscesses may help differentiate aspergillosis from tuberculosis, which typically presents as thin, smooth-walled abscesses. However, fungal infections may share overlapping imaging characteristics, further complicating the ability to reliably differentiate between aspergillosis, candidiasis, and other fungal infections.

A summary of key imaging clues for the diagnosis of various pathogenic causes of infectious spondylitis is provided in Table 1.

**Table 1:** Summary of imaging clues for the diagnosis of various pathogenic causes of infectious spondylitis

Pathogens	Imaging clues	Other clues
Pyogenic	• Affects a single vertebral segment composed of two adjacent vertebrae and the intervertebral disc; most commonly occurs in the lumbar spine • Moderate to severe destruction of the intervertebral disc, with relatively less severe destruction of the vertebral bodies • Epidural and paraspinal abscesses are typically small in size, with thick, irregular, rim-enhancing walls	Insidious onset
Tuberculosis	• Involves multiple vertebral levels (≥3), most commonly in the thoracic spine; subligamentous spread is common • Relative sparing or mild destruction of the intervertebral disc • Severe vertebral destruction can lead to vertebral collapse, kyphosis, and gibbus deformity • Paravertebral, epidural, and paraspinal abscesses are typically large, with thin, smooth, rim-enhancing walls, ± calcification	Indolent onset
Brucellosis	• Simultaneous onset of new bone formation and bone destruction, typically affecting the lumbar spine • Characteristic bone erosion at the anterosuperior vertebral corner (“Pedro Pons’ sign”) • Peri-vertebral new bone resembling anterior osteophytes (“parrot’s beak” appearance)	Occurs in endemic regions linked to consumption of contaminated milk or meat
Melioidosis	• Mimics spinal tuberculosis radiologically	Common in endemic regions (e.g., Southeast Asia, Northern Australia)
Actinomycosis	• Most commonly affects the cervicofacial region • Often results from the spread of nearby infections to the spine • Usually spares the disc	Rare, with limited case reports
Fungal infections	• Less osseous destruction • Relatively preserved disc • Inflammatory mass shows low T2 signal intensity • Small paraspinal abscess	Immunocompromised patients

## Conclusions

Infectious spondylodiscitis, whether caused by pyogenic bacteria, tuberculosis, or other less common organisms, presents diagnostic challenges. Although imaging findings often overlap across spondylodiscitis caused by different pathogens and laboratory or pathologically confirmed diagnosis continues to be the gold standard, imaging, particularly MRI, plays a crucial role in the management and provides clues for the accurate diagnosis. Clinical information and imaging findings are required to guide treatment decisions.

## Data Availability

All data are available as part of the article and no additional source data are required.
